# Antimicrobial Activity of *Smilax china* L. Root Extracts against the Acne-Causing Bacterium, *Cutibacterium acnes,* and Its Active Compounds

**DOI:** 10.3390/molecules27238331

**Published:** 2022-11-29

**Authors:** Ji-Hae Joo, Min-Hui Han, Ja-I Kim, Jong-Eun Kim, Kyung-Hwan Jung, Han Sun Oh, Young Soo Chung, Hyun Jin An, Jae Duk Lee, Gi-Seong Moon, Hyang-Yeol Lee

**Affiliations:** 1 Division of Food Science and Biotechnology, Korea National University of Transportation, Chungju 27909, Chungbuk, Republic of Korea; 2 Beauty Cosmetic Co., Ltd., 274-14 Wonnamsandan-ro, Wonnam-myeon 32740, Eumseong-gun, Chungbuk, Republic of Korea; 3 Yeomyung Biochem Co., Ltd., 7-4 Tabyeon 1-gil, Gangane-myeon, Heungdeok-gu, Cheongju-si 28171, Chungbuk, Republic of Korea

**Keywords:** skin acne, antimicrobial activity, *Smilax china* L., *Cutibacterium acnes*, cytotoxicity

## Abstract

The root of *Smilax china* L. is used in traditional Korean medicine. We found that the *Smilax china* L. root extract has strong antimicrobial activity against two *Cutibacterium acnes* strains (KCTC 3314 and KCTC 3320). The aim of this study was to identify the beneficial properties of *Smilax china* L. extracts for their potential use as active ingredients in cosmetics for the treatment of human skin acne. The high-performance liquid chromatography (HPLC) and liquid chromatography-hybrid quadrupole time-of-flight mass spectrometry (LC/QTOF/MS) methods were used to obtain the profile of secondary metabolites from the ethyl acetate-soluble fraction of the crude extract. Agar diffusion and resazurin-based broth microdilution assays were used to evaluate antimicrobial activity and minimum inhibitory concentrations (MIC), respectively. Among the 24 metabolites, quercetin, resveratrol, and oxyresveratrol were the most potent compounds against *Cutibacterium acnes*. Minimum inhibitory concentrations of quercetin, resveratrol, and oxyresveratrol were 31.25, 125, and 250 μg/mL, respectively.

## 1. Introduction

*Cutibacterium acnes* is a Gram-positive bacterium that is usually found on the surface of human skin and is associated with acne vulgaris [1]. Acne vulgaris is a chronic inflammatory disease of the pilosebaceous unit, and acne bacteria live deep within follicles, pores, and on the surface of healthy skin [2,3]. Therefore, acne is one of the most common skin diseases, predominantly affecting adolescents and young adults. Prescriptions to treat acne are often for antibiotics such as clindamycin [4]; however, antibiotics sometimes cause side effects such as burning, itching, scaliness, and contact dermatitis [5,6]. An antiseptic, benzoyl peroxide, is effective for treating acne lesions and it does not induce antibiotic resistance. Common side effects of benzoyl peroxide [7] include redness, burning, and irritation [8]. A combination of clindamycin and benzoyl peroxide exists in the form of a topical gel used for the treatment of acne [9]; however, all formulations are prescription drugs that are not suitable for daily use products such as cosmetics. Natural products have been popular for decades for developing new cosmetics owing to the relatively high potency and low toxicity of traditional medicinal plants [10,11]. Recently, we found that the extract of *Smilax china* L. root effectively reduced the growth of *C. acnes*. Many secondary metabolites have been isolated from the medicinal plant, *Smilax china* [12,13]. For instance, stilbenes and flavonoids such as oxyresveratrol, resveratrol, and scirpusin A [14,15], steroidal saponins [16,17,18], and cytotoxic phenylpropanoid glycosides were isolated from *Smilax china* [19,20,21,22,23,24,25]. The new compounds, phenylpropanoid glycosides, isolated from the stems of *Smilax china,* are known smilasides [26]. In this study, to identify the active compounds from the crude extract of *Smilax china*, two different strains of *C. acnes* were tested on the fractions of crude extract and the pure compounds isolated from the fractions of the extract. The minimum inhibitory concentration (MIC) and minimum bactericidal concentration (MBC) of the isolated active compounds were tested against two *C. acnes* strains (KCTC 3314 and KCTC 3320).

## 2. Results

### 2.1. Antimicrobial Activity of Extract of Smilax china L. Root

**Figure 1:**
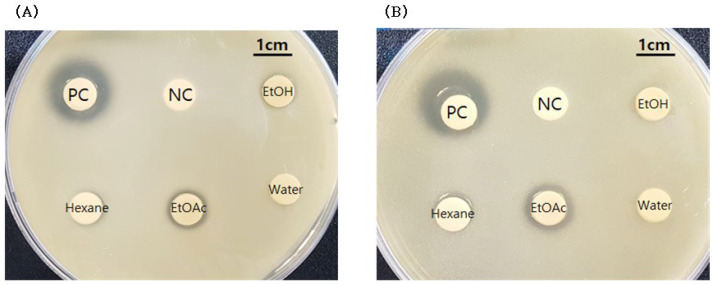
Antimicrobial activity of fractions of *Smilax china* L. root extract. (**A**) *Cutibacterium acnes* KCTC 3314; (**B**) *Cutibacterium acnes* KCTC 3320. PC, positive control (Erythromycin, 5 μg/mL in DMSO); NC, negative control (DMSO, 100%); EtOH, ethyl alcohol-soluble fraction; Hexane, hexane-soluble fraction; EtOAc, ethyl acetate-soluble fraction; Water, water-soluble fraction of *Smilax china* L. root extract.

Based on the agar diffusion method for antimicrobial activity, as shown in Figure 1, only the ethyl acetate soluble fraction of *Smilax china* L. root extract showed activity against *Cutibacterium acnes* KCTC 3314 (10.3 mm in diameter) and KCTC 3320 strains (11.8 mm), and this fraction was used for further analysis.

### 2.2. Analysis for Chemical Compositions of the Ethyl Acetate Fraction from Smilix china L. Root Crude Extract

**Figure 2:**
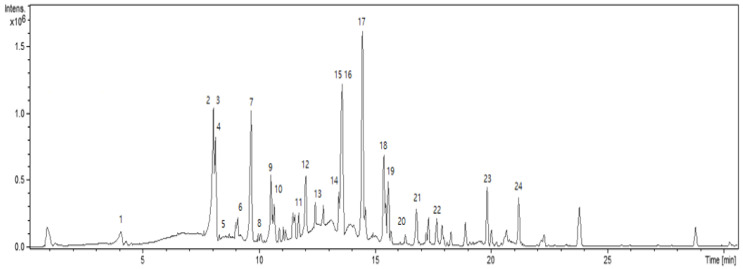
LC/QTOF/MS chromatogram showing natural products from ethyl acetate-soluble fraction of *Smilax china* L. root extract.

First, the chemical compositions of the ethyl acetate fraction were investigated to reveal the major components of the mixture by using high-resolution liquid chromatography-hybrid quadrupole time-of-flight mass spectrometry (LC/QTOF/MS), as shown in Figure 2 and Table 1. Typical phytochemicals, such as organic acids and flavonoids, were identified from the exact mass of each isolated compounds and their theoretical mass. Stilbenoids, such as polydatin, oxyresveratrol, and resveratrol, were also found. The presence of quinic acid, caffeic acid, polydatin, quercetin, oxyresveratrol, catechin, and resveratrol was further confirmed by HPLC using standard compounds.

Unique phenylpropanoid compounds, smilasides [26], were also detected using liquid chromatography-hybrid quadrupole time-of-flight mass spectrometry (LC/QTOF/MS). Peak 22 showed a quasi-molecular ion at m/z 777.2268 [M-H]^−^, which is consistent with the molecular formula C_36_H_42_O_19_ of smilaside A. Peak 23 shows a quasi-molecular ion at *m/z* 839.2405 [M-H]^−^, which is consistent with the theoretical exact mass 839.2399 of the molecular formula C_41_H_44_O_19_ of smilaside C. The detected mass of peak 24 is 881.2525, which is consistent with smilaside D or E, but we could not confirm exactly which one is D or E with only the mass result.

### 2.3. Antimicrobial Effect against Cutibacterium acnes of Fractions 1–5 Obtained from Silica Gel Column Chromatography

Fractions 1–5 of the crude extract were obtained using flash silica gel column chromatography with an eluent of 20% ethyl acetate in hexane as a starting point. The ethyl acetate content in the eluent was then gradually increased up to 50%. Fraction 1 obtained from the eluent with 20% ethyl acetate in hexane and fraction 2 obtained from the eluent with 50% ethyl acetate in hexane exhibited potent antimicrobial activity against *C. acnes* KCTC 3314 and KCTC 3320 strains, with the *C. acnes* KCTC 3314 strain being more susceptible to both fractions. The fractions 3, 4, and 5 obtained from flash column chromatography with eluents of 80% ethyl acetate in hexanes, 100% methylene chloride, and 20% methanol in methylene chloride did not show any antimicrobial activity against *C. acnes,* respectively.

**Figure 3:**
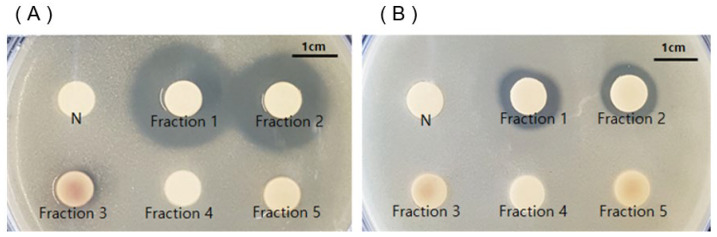
Antimicrobial activity of fractions from silica gel column chromatography to which ethyl acetate extract of *Smilax china* L. root was subjected. (**A**) *C. acnes* KCTC 3314; (**B**) *C. acnes* KCTC 3320. N, negative control (DMSO, 100%); Fractions 1~5, fractions from silica gel column chromatography.

Among the obtained fractions 1–5, only fractions 1 and 2 showed strong inhibition zones against *C. acnes* KCTC 3314 (22.3 and 21.3 mm, respectively) and *C. acnes* KCTC 3320 (13.3 and 12.0 mm, respectively), as shown in Figure 3. In particular, the inhibition zone diameters of fractions 1 and 2 against *C. acnes* KCTC 3314 were larger than those against *C. acnes* KCTC 3320.

### 2.4. Antimicrobial Effect of Isolated Compounds (Quercetin, Resveratrol, and Oxyresveratrol) against Cutibacterium acnes

**Figure 4:**
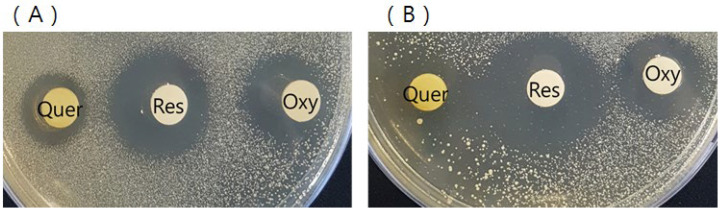
Antimicrobial effect of isolated compounds (Quer; quercetin, Res; resveratrol, and Oxy; oxyresveratrol) against *C. acnes* KCTC3314 (**A**) and *C. acnes* KCTC3320 (**B**).

Among the many identified compounds from the ethanolic extracts, three of them (quercetin, resveratrol, and oxyresveratrol) exhibited the strongest antimicrobial activity against two *C. acnes* strains (KCTC 3314 and KCTC 3320). The antimicrobial properties of compounds 13, 17, and 20 were assessed. The results revealed that the compounds 13, 17, and 20 efficiently suppressed the growth of *C*. *acnes*, which is involved in the pathogenesis of acne. Acne is a common skin disease and predominantly affects adolescents and young adults. The isolated compounds, quercetin, resveratrol, and oxyresveratrol, had strong inhibition zones against *C*. *acnes* KCTC 3314 (17.1, 26.5, and 21.4 mm) and *C*. *acnes* KCTC 3320 (12.3, 25.6, and 18.9 mm), respectively, as shown in Figure 4. Resveratrol showed the strongest antimicrobial activity against both *C*. *acnes* strains, as determined by the agar diffusion test.

Minimum inhibitory concentration (MIC) of the identified active compounds 13, 17, and 20 were measured using a resazurin assay. Minimum inhibitory concentration of quercetin, resveratrol, and oxyresveratrol were 31.25, 125, and 250 μg/mL, respectively. Quercetin is the most potent antimicrobial compound against *C. acnes* KCTC3314, as summarized in Table 2.

The minimum bactericidal concentrations (MBC) of compounds 13, 17, and 20 were measured. The MBCs of compounds 13, 17, and 20 were identical to their MICs, as listed in Table 3.

### 2.5. Cytotoxicity of Compounds 3~5, 11, 13, 17, and 30

**Figure 5:**
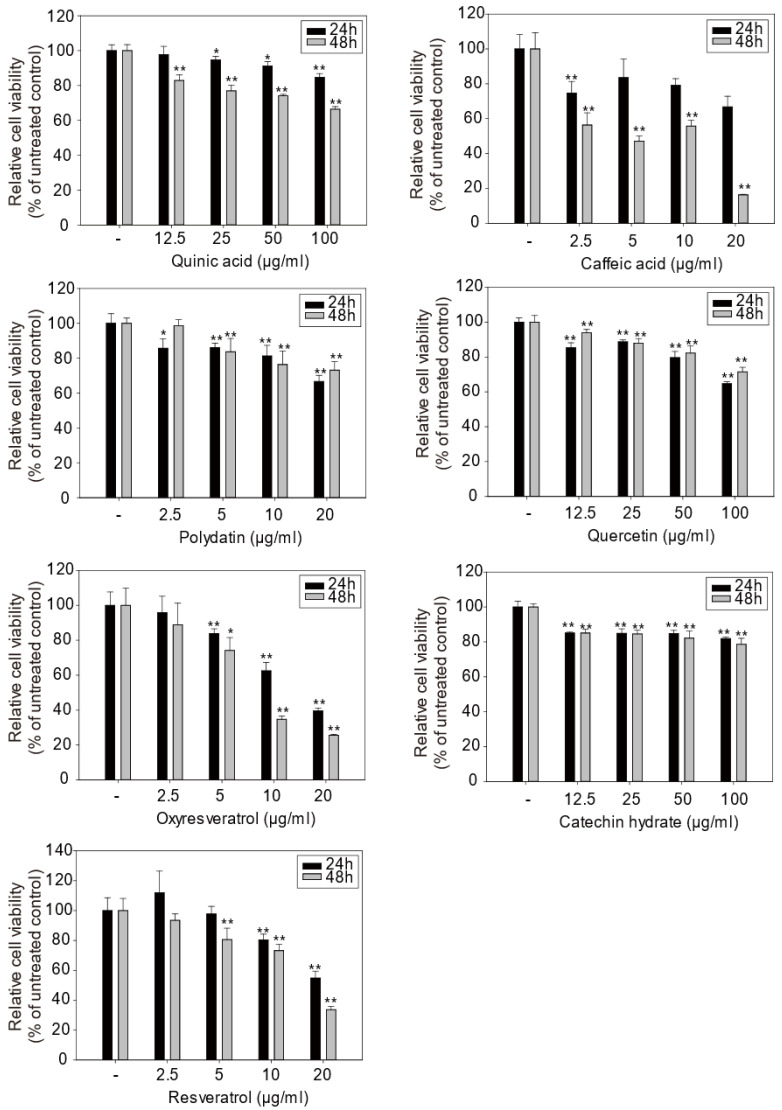
Cell viability of each chemical on skin keratinocyte HaCaT cells. Cell viability was measured using the MTT assay described in Materials and Methods. Data are shown as mean ± SD and asterisks (*) indicate a significant inhibition by each compound compared with the untreated control group (*, *p* < 0.05 and **, *p* < 0.01).

Seven compounds identified from *Smilax china* L. root were examined using the MTT assay to investigate their cytotoxic effect on skin cells. Quinic acid, quercetin, and catechin were not toxic to cells up to a concentration of 100 μg/mL. Resveratrol was also found in grapes [27,28] and showed mild cytotoxicity at a concentration of 20 μg/mL. Oxyresveratrol and caffeic acid were slightly more cytotoxic than resveratrol, which is one of the major components of the *Smilax china* L. root extract, based on the MTT assay. Overall, most of the metabolites of *Smilax china* L. root did not show any significant cytotoxicity up to 10 μg/mL. However, all MICs of *Smilax china* L. root crude extracts against *C*. *acnes* KCTC 3314 were 500 ppm. The mean percentage of the major active compound (resveratrol) contained in the dried *Smilax china* L. root extracts, was 0.35 wt% (our unpublished data). Therefore, final products are anticipated to contain approximately 1.75 ± 0.2 ppm (1.75 μg/mL) of resveratrol for anti-acne activity. Moreover, resveratrol did not show any cytotoxicity at the concentrations of approximately 5 ppm, as shown in Figure 5.

## 3. Discussion

In this study, we investigated the antimicrobial properties of root extracts from *Smilax china* L. against *C. acnes* to determine the potential of this extract for cosmetic applications. A number of studies [12,13] have described the antibacterial properties of *Smilax china* L. leaves and root extracts. However, there is limited information on the use of *Smilax china* L. root extracts for biological applications in the cosmetic industry. 

To identify the active compounds in the extract, the crude *Smilax china* L. extract was separated from an ethyl acetate–water mixture, fractionated by flash silica gel column chromatography, and purified by prep-HPLC. *C*. *acnes* KCTC3314 and 3320 strains were tested for anti-acne activity of fractionated mixtures. Preliminary experiments revealed that the crude *Smilax china* L. root extract had antimicrobial activity against both Gram-positive and Gram-negative bacteria (our unpublished data). Interestingly, crude *Smilax china* L. root extract demonstrated potent anti-acne activity against *C. acnes* as well.

The phenolic compounds found in *Smilax china* L. extracts are known to have varying antimicrobial activities. The synergic action, which has been observed for many phenolic compounds, can significantly enhance the antimicrobial effect [29]. Many polyphenolic compounds isolated from *Smilax china* L. have antimicrobial activity against bacteria such as *Salmonella typhimurium, Listeria monocytogenes, Staphylococcus aureus, Bacillus subtilis, and Escherichia coli* [12,25]. Interestingly, we found that only quercetin, one of many flavonoids isolated from *Smilax china* L. root extract, had a high antimicrobial effect against *C. acnes*, whereas two stillbenoid compounds, oxyresveratrol and resveratrol, had antimicrobial activity against *C. acnes*.

The antimicrobial activity and low toxicity of the extract make it suitable for a wide range of dermatological applications, including the treatment of *acne vulgaris*.

## 4. Materials and Methods

### 4.1. Materials

*Smilax china* L. root used in this study originated from Gyeongju city, Gyeongsangbuk-do, Korea and was supplied by Hwalim Natural Drug Co., Ltd. (Busan, Korea). *C. acnes KCTC* 3314 and KCTC3320 strains were purchased from the Korean Collection for Type Cultures (Jeongeup, Korea) and used for antimicrobial activity tests as human skin acne-causing bacteria. The solvents of Ethyl alcohol (95%), Methylene chloride, Ethyl acetate, n-Hexane, Methyl alcohol (99%), and Trifluoro acetic acid (TFA) were purchased from Samchun Chemical Co., Ltd. (Seoul, Korea). Acetonitrile and Formic acid were purchased from TEDIA (Fairfield, OH, USA) and Daejung Chemical Co. (Goryeong, Korea), respectively. Standard materials of (+)-Catechin hydrate, Chlorogenic acid crystalline, D-Quinic acid (98%), Caffeic acid, Oxyresveratrol, Resveratrol, Polydain, and Quercetin were purchased from Sigma-Aldrich, Inc. (St. Louis, MO, USA).

### 4.2. Preparation of Extract of Smilax china L. Root

**Figure 6:**
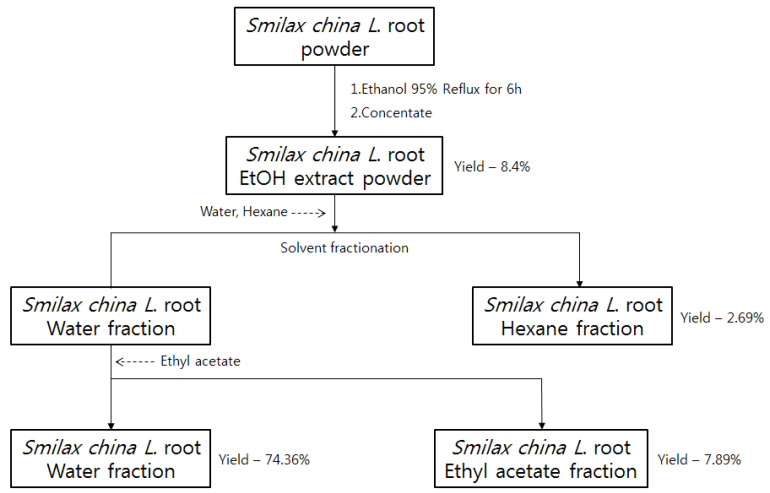
The flowchart of sample preparation.

To extract compounds, 100 g of Smilax china L. root powder was mixed with 95% ethanol in a round-bottom flask equipped with a mechanical stir for 12 h at 25 °C. The mixture was then concentrated to remove ethanol, and 8.40 g of powdered extract was obtained. In total, 8.40 g of the crude extract was treated with n-hexane and distilled water (50:50). The n-hexane layer was evaporated and concentrated (Yield, 2.69%). The water layer was separated with methylene chloride, ethyl acetate, and n-butanol, respectively. Ethyl acetate fraction was then dried over Na_2_SO_4_ and concentrated under reduced pressure to give 0.66 g (yield; 74.36% for water fraction and 7.89% for ethyl acetate fraction out of 8.40 g ethanol extract) of a sample as power for the UPLC/QTOF/MS analysis as shown in Figure 6.

### 4.3. High-Resolution UPLC/QTOF/MS Analysis

Ultra-performance LC/QTOF/MS analysis was performed on an ultra-high-resolution Q-TOF LC-MS/MS system (Micro QTOF III, Bruker Daltonix Gmbh, Bremen, Germany.) using a C18 column (Ace 3 C18-300), with a particle size of 1.7 mm, dimensions of 2.1 mm × 125 mm, and flow rate of 0.4 mL min^−1^, and an electrospray ionization (ESI) source. The eluent solvents consisted of 0.1% formic acid in water (eluent A) and 0.1% formic acid in acetonitrile (eluent B). The mobile phase consisted of (A) and (B) with a gradient elution of 0–55% B at 0–30 min and 55–95% B at 30–40 min.

### 4.4. HPLC Analysis

The extracts were analyzed by high-performance liquid chromatography (HPLC, YL 9112S) equipped with PDA detector and a reverse-phase C18 column (Kinetex, 4.6 mm × 250 mm, 5 μm, 100 Å). The flow rate applied was 1.0 mL/min. Pure compounds were isolated with prep-HPLC equipped with a reverse-phase C18 column (YMC-Triart, 10 mm × 250 mm, 5 μm, 12 nm) and the flow rate applied was 2.5 mL/min. The eluent solvents consisted of 0.1% trifluoroacetic acid in water (eluent A) and 0.1% trifluoroacetic acid in acetonitrile (eluent B). The mobile phase consisted of (A) and (B) with a gradient elution of 0–25% B at 0–30 min, and 25–45% B at 30–40 min. The detection wavelengths were set at 220 and 300 nm.

### 4.5. Preliminary Antimicrobial Activity

The agar diffusion method was used to confirm the antibacterial activity of *Smilax china* L. root extract (conc. 10 mg/mL of DMSO), its fractions (conc. 10 mg/mL of DMSO), and pure components (conc. 10 mg/mL of DMSO) including resveratrol, oxyresveratrol, and quercetin against acne-causing *C. acnes* [30]. *C. acnes* KCTC 3314 and KCTC 3320, which are strictly anaerobic bacteria, were cultured in RCM broth (BD, Sparks, MD, USA) in an anaerobic jar (Oxford, Cambridge, UK) with GasPaK™ (BD). The culture was inoculated in RCM soft agar (0.7% agar, *w/v*) and poured onto an RCM agar plate. Disc paper with 20 μL of each sample (0.2 mg/disc) was loaded on the plates and incubated at 37 °C for 24 h, anaerobically described above and inhibition zones around discs were examined.

### 4.6. Determination of Minimum Inhibitory Concentrations and Minimum Bactericidal Concentration

To measure minimum inhibitory concentration (MIC) of antimicrobial compounds including resveratrol, oxyresveratrol, and quercetin, a resazurin assay was performed as per a previous study with modifications, since DMSO, the solvent for the compounds, was toxic to *C. acnes* KCTC 3314 and the limited volume of diluted samples in DMSO was acceptable not to inhibit its growth [31]. Namely, in 96-well microplates, 20 µL of 10^6^ CFU/mL of *C. acnes* KCTC 3314 was inoculated in 176 µL of RCM broth to be 10^5^ CFU/mL in the final volume of 200 µL and each 4 µL of serially 2-fold diluted sample in DMSO, which ranged from 1000 to 7.8125 ppm in the final volume and was added in triplicates. The microplate was incubated at 37 °C for 24 h in an anaerobic jar (Oxford, UK) with GasPaK™ (BD). After incubation, 20 µL of resazurin (1 mg/mL) were added to each well of the microplate and incubated at 37 °C for 4 h in an orbital shaker with 100 rpm for resazurin metabolization. Then, the color change was examined. MIC was determined as the lowest concentration where the color was not changed. To determine minimum bactericidal concentration (MBC), the above diluents for MIC test were used. The volume of two microliters from each diluent was plated on a RCM agar plate. The MBC was determined by the lowest concentration of compound at which no bacterial colony of *C. acnes* KCTC 3314 was shown on the plate, which means the MBC value reduced initial viable cell counts by ≥99.9% [32,33,34].

### 4.7. Cell Viability Assay

Cell viability of HaCaT cells was determined by an MTT assay [35,36]. HaCaT cells were cultured in the 96-well plates at 5.0 × 10^4^ cells per wells and incubated in completed DMEM. Cells were starved in serum-free DMEM for 12 h. After serum-starvation, the cells were incubated with different concentrations of each chemical. After the addition of MTT solution (10% *v/v*) in serum-free DMEM, the cells were incubated for 1 h. The medium was removed and dimethyl sulfoxide (DMSO) was added to each well to dissolve formazan crystals. The absorbance at 570 nm was measured using a microplate reader.

## 5. Conclusions

Taken together, we successfully extracted components from the roots of *Smilax china* L. and studied them against Gram-positive *C. acnes* strains, which are involved in skin diseases such as acne. Based on UPLC/QTOF/MS and silica gel column chromatography, three active components were selected, quercetin, resveratrol, and oxyresveratrol. The components showed strong inhibition zones against *C*. *acnes* KCTC 3314 and KCTC 3320 strains. Among these components, resveratrol greatly inhibited the growth of both strains followed by oxyresveratrol and quercetin, respectively. However, quercetin showed the lowest MIC and MBC values of 31.25 ug/mL against *C. acnes* KCTC 3314 strain and was found to have lower cytotoxic activity compared with resveratrol and oxyresveratrol. To the best of our knowledge, this is the first study to identify the active ingredients of *Smilax china* L. root extracts and investigate their antibacterial effects against *C. acnes*. 

To further investigate the potential of *Smilax china* L. root extracts for cosmetic applications, various types of cosmetic products need to be produced and tested for clinical trials in the near future.

## Figures and Tables

**Table 1:** Compounds identified from ethyl acetate-soluble fraction of *Smilax china* L. root extract.

No.	RT (min)	Compound Name	Formula	Detected Mass(M-H) *	Theoretical Exact Mass(M-H)
		*Phenols*			
1	4.3	1,2-Benzenediol	C_6_H_6_O_2_	109.0294	109.0290
		*Acids*			
2	8.1	Chlorogenic acid	C_16_H_18_O_9_	353.0878	353.0873
4	8.2	Quinic acid	C_7_H_12_O_6_	191.0560	191.0556
5	8.3	Caffeic acid	C_9_H_8_O_4_	179.0352	179.0350
		*Flavonoids*			
3	8.1	Catechin	C_15_H_14_O_6_	289.0719	289.0712
6	9.5	Afzelechin	C_15_H_14_O_5_	273.0768	273.0763
7	9.7	Leucopelargonidin	C_15_H_14_O_6_	289.0714	289.0712
8	9.9	Astilbin	C_21_H_22_O_11_	449.1078	449.1084
10	10.7	Procyanidin B3	C_30_H_26_O_12_	577.1359	577.1346
14	13.4	Kaempferol 7-O-β-D-glucopyranoside	C_21_H_20_O_11_	447.0938	447.0927
12, 15, 18	12.0, 13.6, 15.4	Cinchonain Ia~c	C_24_H_20_O_9_	451.1040	451.1029
16	13.7	Engeletin	C_21_H_22_O_10_	433.1129	433.1135
20	16.2	Quercetin	C_15_H_10_O_7_	301.0365	301.0354
		*Stillbenoids*			
11	11.7	Polydatin	C_20_H_22_O_8_	389.1248	389.1236
13	12.4	Oxyresveratrol	C_14_H_12_O_4_	243.0665	243.0657
17	14.5	Resveratrol	C_14_H_12_O_3_	227.0715	227.0708
21	16.8	Scirpusin A	C_28_H_22_O_7_	469.1281	469.1287
		*Phenylpropanoids*			
9	10.5	Cinchonain IIa	C_39_H_32_O_15_	739.1670	739.1663
19	15.6	Helonioside A	C_32_H_38_O_17_	693.2051	693.2031
22	17.7	Smilaside A	C_36_H_42_O_19_	777.2268	777.2242
23	19.8	Smilaside C	C_41_H_44_O_19_	839.2405	839.2399
24	21.2	Smilaside D or E	C_43_H_46_O_20_	881.2525	881.2504

* Detected on ESI^-^ mode. No represents the number of each peak from LC/QTOF/MS chromatogram in Figure 2. RT represents retention time on LC/QTOF/MS chromatogram.

**Table 2:** Minimum inhibition concentration (MIC) of active compounds 13, 17, and 20 against *C. acnes* KCTC 3314.

No.	Compounds	MIC (μg/mL)
13	Oxyresveratrol	250
17	Resveratrol	125
20	Quercetin	31.25
	Clindamycin ^1^	0.0625

^1^ Positive control.

**Table 3:** Minimum bactericidal concentration (MBC) of active compounds 13, 17, and 20 against *C. acnes* KCTC 3314.

No.	Compounds	MBC (μg/mL)
13	Oxyresveratrol	250
17	Resveratrol	125
20	Quercetin	31.25
	Clindamycin ^1^	0.0625

^1^ Positive control.

## Data Availability

Date are available on request from the corresponding author.

## Sample Availability

Samples of the compounds are not available from the authors.
